# 

**Published:** 1882-12

**Authors:** 


### INDEX

TO THE

### HOMŒOPATHIC PHYSICIAN.

### VOLUME II.

PAGE.
Abdomen, Bearing down Pains, Pressure, etc., in. E. J. L. .......... 34
Aconitum nap. .......... 82, 84, 101, 228, 358, 387, 388, 389
Actea rac. .......... 34
Address before the American Institute, by Dr. Breyfogle, June 13th, 1882. Ad. Lippe, M. D. .......... 447
Agaricus m. .......... 34, 82, 230
Agnus c. .......... 270
Ague and Quinine. E. J. L. .......... 263
Aloe. .......... 32, 82, 190, 271
Alternation of Remedies. Editorial... 9
Alumina. .......... 347
Ambra gr. .......... 82
American Institute. Session of 1882. P. P. Wells, M. D. .......... 361
The American Joker. .......... 136
Ammonium Carb. .......... 82, 145
Amygdalis p. .......... 52
Anacardium. .......... 231
Anæmia, Progressive Pernicious. E. Cranch, M. D. .......... 43
Antimonium cr. .......... 34, 45
Antimonium tart. .......... 303, 304, 497, 498
Appendix, Hæmorrhoids. By W. Jefferson Guernsey, M. D. .......... 1-25
Index of Symptoms of the Respiratory Organs. Drs. Clark and Lee. 1-40
Aphis Chenopodii glauci. .......... 145
Apis mel. .......... 34, 98, 380, 438
Apis mel. Its Poison. Jas. Hedden... 185
Aranea d. .......... 399
Argent. nit. .......... 34
Arnica. .......... 189, 358
Arsenic .......... 44, 51, 83, 142, 147, 190, 277, 305, 354, 380, 381, 401, 468
Arum tri. .......... 98, 303, 467
Arum tri. in Sequelæ of Typhoid Fever. E. W. Berridge, M. D. .......... 467
Asafœtida. .......... 34, 239
Asarum euro. .......... 83
Attention, Central Ohio Hom. Med. Society. .......... 416

Badiaga. .......... 152
Baer, O. P., M. D. Infantile Convulsions. .......... 381
Ballard. E. A., M. D. Dyspnœa on Falling Asleep. .......... 303
Bayard, Edward. M. D. The Obligations of the World to Hahnemann.. 201

PAGE
Belladonna. .......... 34, 53, 83, 93, 274, 384, 389, 401, 443, 469
Bell, J. B., M. D. Senile Gangrene. Amputation of Thigh. .......... 353
Berridge, E. W., M. D. Case of Puerperal Fever followed by Phlegmasia Alba Dolens. .......... 388
Clinical Cases. .......... 47, 90, 466
Euthanasia in Phthisis. .......... 138
Berberis. .......... 83, 239, 504, 506
Berberis in Colic and Inflammatory Stricture. G. N. Brigham, M. D. ...... 506
Better after Sleep. C. Hering (extract). 477
Books Noticed:
Special Pathology and Diagnostics, by C. G. Rawe, M. D. .......... 54
Insanity and its Treatment, by Samuel Worcester, M. D. .......... 55
Proceedings of the Hom. Med. Society of Ohio. .......... 56
Journal of European Travels. C. Pearson, M. D. .......... 56
New Books. .......... 56
Ophthalmic Therapeutics, by Geo. S. Norton, M. D. .......... 102
Ophthalmoscope, by C. H. Vilas, M. D. .......... 103
The Child of Promise, etc., by W. M. Cate. .......... 104
Visiting List. Otis Clapp & Sons...... 104
Treatise on Mat. Med. Cowperthwaite. .......... 198
The Human Ear. Winslow. .......... 199
Bureau of Sanitary Science; Report of Am. Inst. of Hom. .......... 199
Scratches of a Surgeon. Helmuth.. 199
Electricity. Boyce. .......... 199
Diseases of the Eye, by H. C. Angell, M. D. .......... 286
Electricity in Surgery, by John Butler, M. D. .......... 287
Leucorrhœa, etc., by A. M. Cushing, M. D. .......... 287
Smith's New Label Holder. .......... 287
Supersalinity of Blood. Burnett.... 320
American Pharmacopœa. .......... 320
Diet of Infants and Young Children, by J. C. Morgan, M. D. .......... 359
Domestic Practice, by M. M. Eaton, M. D. .......... 359
Address before American Institute, 1882. .......... 359

Books Noticed (*Continued*). PAGE
Diseases of the Pancreas and their Homœopathic Treatment........... 360
American Medicinal Plants, by C. F. Millspaugh ........................... 404
Hand-book of Diseases of the Heart 404
Phthisis Pulmonalis, or Tubercular Phthisis, by G. N. Brigham, M. D............................................ 440
Doctor, What Shall I Eat? by Chas. Gatchell, M. D........................... 471
Observations on Therapeutic Use of Alcohol, by A. K. Hills, M. D.. 471
Address by the President of the Hom. Med. Society of Penna...... 471
Book Reviews, Notices, etc., 54, 55, 56, 102-3-4, 286-7, 359-60, 404, 440, 471
Bœnninghausen's Experience with High Potencies............................ 477
Bœnninghausen on Motion as a Cause of Agg. (extract)........................ 479
Borax. ..............................................83, 96
Bovista.............................................34, 83
Brewster, A. J., M. D., High Potencies in Acute as well as Chronic Diseases. 273
Brigham, G. N., M. D., Berberis in Colic and Inflammatory Strictures.......... 506
Bromine .............................................83, 276
Bruns, F., M. D., Lyc. in Bronchitis...... 470
Bryonia..................52, 83, 98, 99, 140, 143, 231
Bufo ..................................................83, 439
Butler, C. W., M. D., Lycopodium Clinical Cases........................................ 465
Rheumatism—Lac. Can.............. 507

Cactus gr............................................... 83
Calc. carb.......52, 83, 99, 386, 397, 401, 413, 443
Calcarea silicata.......................................... 52
Calcarea phos.......................................... 34
Cantharis......................................34, 83, 354
Cannabis Ind .......................................... 152
Capsicum ............................................... 145
Carbo an ......................................34. 83, 380
Carbo veg...................45, 51, 84, 231, 397, 504
Carcinoma, L. B. Wells. M. D.......... ..... 379
Carleton, C, M. D. A Case of Perineal Abscess ............................................ 502
Cases from Practice. J. B. Gregg Curtis, M. D............................................ 401
Case from Practice. E. P. Hussey, M.D. 276
Case from Practice. C. F. Millspaugh, M. D............................................ 188
Cases from Practice, with Remarks. C. Pearson, M. D................................ 398
Cases. E. P. Rushmore, M. D................ 318
Castoreum.............................................. 239
Catarrh, Acute, Cimicifuga in its Relation to. C. Carleton Smith, M D... 211
Caulophyllum .....................................34, 213
Causticum..........................................45, 84, 468
The Central N. Y. Hom. Med. Society, Proceedings of....................116, 259, 493
Chamomilla..................................34, 53, 54, 84
Checking Pulmonary Hæmorrhage (extract.) ........................................... 485
Chelidonium........................................84. 99, 145
Chest Symptoms, Table of Dr. Hering... 233
China................................34, 84, 355, 391, 392
Chinin sulph............................................ 464
Cimicifuga in its Relation to Acute Catarrh. C. Carleton Smith, M. D...... 211
Cina...........................................52, 496, 497
Clark, George H., M. D. Index of Symptoms of Respiratory Organs......... 278
Norton's Ophthalmic Therapeutics 316
Clausen, D. W., M. D. Pure Homœopathy, Progressive Homœopathy, and The True Homœopathician..... 485

PAGE
Clinical Bureau of Am. Inst. of Homœopathy. T. F. Pomeroy..............32, 485
Clinical Cases. E. W. Berridge, M. D. 47, 90, 466
C. W. Butler, M. D............................ 465
George G. Gale, M. D...................... 468
E. P. Gregory, M. D.......................... 464
C. Lippe, M. D.................................. 190
J. W. Thompson, M. D...................... 469
Clinical Reflections. Ad. Lippe, M. D. 45, 304, 347
Clinical Reports, Value of. E. J. L...... 137
Clinical Reports, with Cases. E. Rushmore, M. D........................................ 144
Cocculus........................................34, 84, 95
Coffea...........................................53, 84, 403
Colchicum.......................................238, 304
Colocynthis.................................34, 51, 469
Conium ................................................. 34
Convulsions, Infantile. O. P. Baer, M. D............................................. 381
Cough Symptoms. Appendix. Drs. Clark and Lee...................................1-40
Cough Symptoms, Table of. Dr. Hering.............................................. 233
Cough, with Vomiting. E. B. Nash, M. D.............................................. 231
Cranch, Edward. Progressive Pernicious Anæmia.................................. 43
Creasote. See Kreasotum.
Criticisms on the Treatment of the late President (extracts)............................ 36
Crocus............................................84, 443
Cundurango.......................................... 150
Cuprum ............................................... 35
Custis, J. B. Gregg, M. D. Cases from Practice........................................... 401
Cutaneous Eruptions caused by the use of Certain Medicines................. 130
Cyclamen............................................. 84

Daphne ind.......................................... 231
Deady, Charles, M. D. Norton's Ophthalmic Therapeutics........................ 268
Diarrhœa Symptoms, Table of. C. Hering, M. D.................................... 136
Digitalis..........................................35, 142
Diphtheria a Disease of the Liver. Dr. Reiter (extract)................................ 505
Discussions in Homœopathic Societies and Treatment of Intermittent Fevers. P. P. Wells, M. D.............. 289
Dose, Repetition of. C. Lippe, M. D.... 500
——————— E. B. Nash. M. D. 114
Drosera rotund.............................35, 145, 231
Duties of the Hour, The. C. Pearson, M. D............................................ 323
Dyspnœa on Falling Asleep. E. A. Ballard, M. D.................................... 303
Editorials:
Alternation of Remedies.............. 9
Homœopathy or Eclecticism..... 321
I. H. Association........................ 153
The Past and the Future ........... 473
Which?........................................ 241
Why We Fail............................... 57
Erysipelas, Rhus in............................. 47
Et Tu, Brute! (extract)........................ 472
Eupatorium........................................ 52
Eupatorium perf...............................88, 89
Euthanasia in Phthisis. E. W. Berridge, M. D........................................ 138

Fatal Errors. Ad. Lippe............14, 75, 433
Fellger, Ad., M. D. Letter from............ 233
Letter of Prof. Butlerow to Prof. Jaeger ........................................... 176

PAGE

Ferrum ......35, 85, 99, 231
Fincke, B., M. D. The Centesimality of the Fincke High Potencies... 213
Neural Analysis...... 169
Homœopathic Infinitesimals are no longer Infinitesimals, but only Minutules...... 453
Potentiation Makes the Medicine Homœopathic...... 417
Provings of Lachesis...... 298
Fistula Lachrymalis and Mylopetroleum Ointment. C. F. Nichols, M. D...... 349
Fluoric acid...... 352
Fluxion Dilutions. Eldridge C. Price, M. D...... 174
Fluxion Potencies. Thos. Skinner, M. D...... 184
Fresh Air: A Necessity. Dr. Oswald (extract)...... 461

Gambogia...... 401
Gale, Geo. G., M. D. Clinical Cases...... 468
Gangrene, Senile. Amputation of Thigh. J. B. Bell, M. D...... 353
Gelsemium......35, 53
Genito-Urinary Therapeutics, Notes on. E. J. L......228, 270
Gnaphalium...... 191
Graphites......35, 303, 356
Gregg, R. R., M. D. Pasteur's Experiments...... 121
Review of Dr. Brigham's Work on Phthisis...... 440
Gregory, E. P., M. D. Clinical Cases... 464
Guernsey, Wm. Jefferson, M. D. A Law of Nature...... 113
Hæmorrhoids. Appendix......1–25

Hæmorrhoids. A. McNeil, M. D...... 437
Hæmorrhoids. W. J. Guernsey, M. D. Appendix......1–25
Hahnemann's Birthday: The Banquet of the Lippe Society...... 225
Hahnemann, The Obligations of the World to. Edward Bayard, M. D... 201
Hahnemann's Opinion, Similia not Idem...... 50
Hall, John, M. D. A Contribution to Surgical Therapeutics...... 350
Haynes, J. R., M. D. Surgical Therapeutics...... 357
Headache, Before, During, and After Menses, by Dr. Minton......193–198
Heddon, James. Apis Mellifica: Its Poison...... 185
Helonias......35, 85
Hellebore...... 401
Hempel, Charles Julius, M. D. A Plea for High Potencies...... 234
Hepar, s. c......35, 85, 100, 101, 192
Hering's Analytical Therapeutics. P. P. Wells, M. D...... 247
Hering, C. Table of Diarrhœa Symptoms...... 136
Tables of Throat, Chest, and Cough Symptoms...... 233
High Potencies in Acute as well as Chronic Diseases. A. J. Brewster, M. D...... 273
High Potencies, The Centesimality of the Fincke. B. Fincke, M. D...... 213
High Potencies, How to Use. Stapf...... 73
High Potencies, A Plea for. Charles Julius Hempel, M. D...... 234
Hint, A Gentle...... 135

PAGE

Homœopathic Infinitesimals are no longer Infinitesimals, but only Minutules. B. Fincke, M. D...... 453
Homœopathic Medical Society of Central New York......116, 493
Homœopathy or Eclecticism? Editorial...... 321
Homœopathy, A Few Thoughts on the Study and Practice of. David Wilson, M. D......219, 250
Homœopathy in Public Hospitals...... 78
Homœopathy, Pure, Progressive Homœopathy, and The True Homœopathician. D. W. Clausen, M. D... 485
Homœopathic Therapeutics and Pathological Anatomy. P. P. Wells...... 18
Homœopathic Society of Central New York...... 259
Hughes, Dr.. Address of before London (1881) Convention. P. P. Wells, M. D...... 158
Hussey, E. P., M. D. Case from Practice...... 276
Hygienic Aphorisms...... 41
Hyoscyamus......35, 52, 85, 275, 385, 387
Hypericum......35, 358

Ignatia...... 85
Index of Cough Symptoms...... 233
I. H. Association, The. Editorial...... 153
International Hahnemannian Association. Officers and Bureaus, 1882... 152
International Hahnemannian Association, Meeting of...... 277
International Hahnemannian Association, The Second Annual Meeting of...... 372
International Hahnemannian Association, Roll of Members of...... 377
Intermittent Fever. C. F. Millspaugh.. 88
Intermittent Fever, Discussions in Homœopathic Societies and Treatment of. P. P. Wells, M. D...... 289
Iodium......35, 85
Ipecac......85, 192, 231, 238

Jaborandi...... 53
James, W. M., M. D...... 240

Kali bich......35, 85, 238, 276
Kali bromatum...... 101
Kali carb......35, 52, 85, 231, 394, 396, 437
Kreosotum......35, 84, 389

Lac caninum......98, 469, 507
*Lancet, The.* Describes the Homœopathic Physician...... 474
Lachesis......35, 51, 85, 98, 99, 190, 303, 354, 356, 392, 460
Lachesis and Lycopodium in Diphtheria...... 460
Lachesis, Provings of. B. Fincke, M. D...... 298
Lapis alb...... 380
Latent Medication. P. P. Wells, M. D.. 105
Laurocerasus...... 85
Law of Cure, The: The Guide. Dr. Dunham (extract)...... 470
Ledum......85, 272
Ledum in Cutaneous Affections. L. B. Wells, M. D...... 272
Lee, E. J., M. D. Index of Cough Symptoms...... 278
Letter of Prof. Butlerow to Prof. Jaeger, Ad. Fellger, M. D...... 176
Letter from Dr. Fellger...... 233

PAGE
Lilium... 35
Linnell, J. E., M. D. Supra-Orbital Neuralgia... 467
Lippe, Ad, M. D. Clinical Reflections...45, 304, 347
The Address before the American Institute by Dr. Breyfogle, June 13th, 1882... 447
Fatal Errors...14, 75, 232, 433
Misrepresentation...62, 110, 156, 243
Plagiarism... 474
Recognition, The... 368
Tilia Europea... 188
Lippe, C., M. D. Clinical Cases... 190
Repetition of the Dose... 500
Lippe Society, Banquet of the, Hahnemann's Birthday... 225
Lobelia inf...35, 85
Lycopodium...35, 53, 85, 143, 145, 192, 380, 393, 394, 402, 460, 465, 466, 470
Lyc. in Bronchitis. F. Bruns, M. D... 470

McNeil, A., M. D. Hæmorrhoids... 437
Magnesia carb...402, 403
Magnesia mur...86, 239
Magnesia phos... 320
Malandrinum... 50
Management of the Specific Remedy. P. P. Wells, M. D... 405
Materia Medica and its Practical Uses. P. P. Wells, M. D... 335
Medical Society of Ohio, The Homœopathic... 224
Members of the International Hahnemannian Association, Roll of... 377
Menstrual Headache...193-8
Menstruation, Early and Profuse: Remedies for. E. J. L...82, 83, 84, 85, 86, 87, 88
Menyanthes... 460
Mercurius...35, 86, 443
Merc. iod... 212
Mezereum...231, 395
Microscopic Falsehoods... 379
Millefolium... 86
Millspaugh, C. F., M. D. A Case from Practice... 188
American Medicinal Plants... 404
Misrepresentation. Ad. Lippe, M. D..62, 110, 205, 243
Moore, Dr. Thomas. Obituary... 200
Morphia: Hypodermically. J. Graball 463
Moschus...86, 239
Motion as a Cause of Aggr. Bœnninghausen (extract)... 479
Muriatic acid... 86

Naja trip... 51
Nash, E. B., M. D. Cough, with Vomiting... 231
Repetition of Dose... 114
Natrum bicarb... 192
Natrum carb...35, 86, 318
Natrum mur...35, 46, 52, 86, 90, 145, 403
Natrum sulph... 190
Nature, A Law of. Wm. Jefferson Guernsey, M. D... 113
Neural Analysis. B. Fincke, M. D... 169
Nichols, C. F., M. D. Fistula Lachrymalis... 349
Nitric acid...35, 89, 393
Notes and Notices...240, 288, 472
Notes from our Scrap Book. E. J. L..33, 82
Norton's Ophthalmic Therapeutics. Chas. Deady, M. D... 268
Geo. H. Clark, M. D...102, 317

PAGE
Nux juglans... 86
Nux mosch...36, 86, 239
Nux vom...36, 86, 145, 304, 319, 444

Obituary, Dr. Thomas Moore... 200
Obligations of the World to Hahnemann, The. Edward Bayard, M. D. 201
Oleander... 318
Ophthalmic Therapeutics, Norton's. Geo. H. Clark, M. D... 316
Opium...238, 386

Pamphlets received... 199
Paris quad... 460
Pasteur's Experiments. R. R. Gregg, M. D... 121
Pathological Materia Medica. Dr. Dunham (extract)... 446
Pathological Prescribing *vs.* Specific Prescribing. P. P. Wells, M. D... 66
Pearson, C., M. D. Cases from Practice, with Remarks... 398
The Duties of the Hour... 323
Perineal Abscess, A Case of. C. Carleton, M. D... 502
Periscope...50, 98, 146, 192, 238
Phosphorus...44, 45, 86, 91, 101, 395, 444
Phos. ac...86, 231
Phlegmasia Alba Dolens, Case of Puerperal Fever followed by. E. W. Berridge, M. D... 388
Phthisis, Euthanasia in. E. W. Berridge... 138
Physical Education Fallacies (extract) 78
Phytolacca dec...86, 468
Picric acid...45, 231
Plagiarism. Ad. Lippe, M. D... 474
Platina...36, 86, 235
Plumbum... 36
Podophyllum... 36
Pomeroy, T. F. Clinical Bureau of Am. Inst. of Homœopathy... 32
That Resolution of the Institute.. 435
Potentiation makes the Medicine Homœopathic. B. Fincke, M. D... 417
Price, Eldridge C. Fluxion Dilutions.. 474
Progressive Pernicious Anæmia. Edward Cranch... 43
Provings of Lachesis. B. Fincke, M. D. 298
Psorinum...303, 469
Pulsatilla...36, 53, 97, 146, 355, 389, 390, 468
Puerperal Fever, Case of, followed by Phlegmasia Alba Dolens. E. W. Berridge, M. D... 388

Quinine, Ague and. E. J. L... 263

Ranunculus bulb...303, 403
Rare Symptom. C. Hering (extract)... 485
Ratanhia... 87
Recognition, The. Ad. Lippe, M. D... 368
"Regular's" Opinion of the Pseudo-Homœopath, A. Dr. Cathell... 445
Remedy, The Single. P. P. Wells, M.D... 307
Repertory of Menstrual Headache, by Dr. Minton... 193
Resolution of the Institute, That. T. F. Pomeroy, M. D... 435
Rheum... 36
Rheumatism and Lac. can. C. W. Butler, M. D... 507
Rhododendron... 87
Rhus rad... 49
Rhus tox...36, 47, 48, 53, 87, 98, 232, 319
Royal College of Physicians, Recent Resolution by the... 73

PAGE
Rushmore, E., M. D., Cases .......... 318
Clinical Reports, with Cases .......... 144
Ruta .......... 359, 465

Sabadilla .......... 232
Sabina .......... 36, 87
Sanguinaria canad .......... 87, 148, 192
Sciatica, Gnaphalium in. S. Swan, M. D .......... 191
Scientific Practice (extract) .......... 72
Secale corn .......... 87, 353, 396
Sepia .......... 36, 87, 232, 238, 444
Silicea .......... 36, 87, 100, 146, 238, 319, 380, 466
Silicea Headache. Dr. Dunham. (Extract.) .......... 459
Similia not Idem. Hahnemann's Opinion .......... 50
Skinner, Thomas, M. D. Fluxion Potencies .......... 184
Smith, C. Carleton, M. D. Cimicifuga in its Relation to Acute Catarrh .......... 211
Societies, Discussions in Homœopathic, and Treatment of Intermittent Fever. P. P. Wells, M. D .......... 289
Specific Prescribing as Against Pathological Prescribing. P. P. Wells .......... 66
Specific Remedy, Management of the. P. P. Wells, M. D .......... 405
Specifics. E. J. L .......... 457
Spigelia .......... 460
Spongia tost .......... 87, 101, 142, 143
Stannum .......... 87, 140
Staphysagria .......... 87
Stramonium .......... 87, 238
Sulphur .......... 36, 51, 58, 87, 141, 149, 150, 190, 320, 393, 505
Sulphur iodatum .......... 99
Sulphur and Malaria (extract.) .......... 479
Sulph. ac .......... 88, 96
Supra-orbital Neuralgia. J. E. Linnell, M. D .......... 467
Surgical Therapeutics, A Contribution to. John Hall, M. D .......... 350
Surgical Therapeutics. J. R. Haynes, M. D .......... 357
Swan, S, M. D. Gnaphalium in Sciatica .......... 191
Syphilinum .......... 139, 303, 304
Syphilinum, Proving of. E. W. Berridge .......... 77
Symphytum .......... 355, 359

Tabacum .......... 238

PAGE
Tart. emet. (see Ant. t.) .......... 232
Terebinthina .......... 97
Theridion .......... 237
Thigh, Amputation of. J. B. Bell, M. D .......... 353
Thompson, J. W., M. D. A Clinical Case .......... 469
Throat Symptoms, Table of. Dr. Hering .......... 233
Thuja .......... 52, 146, 398
Trombidium .......... 319
Tuberculinum .......... 402

Unification: A Fatal Error .......... 14
Urtica Urens .......... 148
Ustilago .......... 36

Vaccination. E. J. L .......... 131
Valeriana .......... 239
Veratrum alb .......... 88, 238
Viburnum op .......... 54

Which? Editorial .......... 241
Why We Fail. Editorial .......... 57
Wells, L. B., M. D. Carcinoma .......... 379
Ledum in Cutaneous Affections .......... 273
Wells, P. P., M. D. The American Institute, Session of 1882 .......... 361
Discussions in Hom. Med. Societies and the Treatment of Intermittent Fever .......... 289
Hering's Analytical Therapeutics .......... 247
Homœopathic Therapeutics and Pathological Anatomy .......... 18
Dr. Hughes's Address before the London Hom. Convention (1881) .......... 158
Latent Medication .......... 105
Management of the Specific Remedy .......... 405
Materia Medica and Its Practical Uses .......... 335
The Single Remedy .......... 307
Specific Prescribing as against Pathological Prescribing .......... 66
Wilson, David, M. D. A Few Thoughts on the Study and Practice of Homœopathy .......... 219, 250

Zincum .......... 88, 238
Zingiber .......... 88, 238

VOL. II. DECEMBER, 1882. No. 12.

### THE HOMŒOPATHIC PHYSICIAN,

### A MONTHLY JOURNAL OF MEDICAL SCIENCE.

"*Magna est veritas, et prævalebit.*"

E. J. LEE, M. D.,
EDITOR.

CONTENTS:

(See inside of Cover.)

NOTICE.

***All articles for publication, books for review, business communications, etc., must be sent to Editor, 2109 Chestnut Street, Philadelphia.***

PHILADELPHIA:

No. 2109 CHESTNUT STREET.

NEW YORK:
A. L. CHATTERTON PUBLISHING COMPANY, PUBLISHER'S AGENTS.

LONDON:
ALFRED HEATH & CO., SOLE AGENTS FOR GREAT BRITAIN,
114 Ebury Street, Eaton Square.

***Yearly Subscription, $2.50, invariably in advance.***

Entered at the Philadelphia Post-Office as Second Class Mail Matter.

### ATTENTION!

"*Resolved*, That it is the opinion of the publishers of THE HOMŒOPATHIC PHYSICIAN that no journal can properly sustain the responsibilities or fulfill all the duties of its position unless it enjoys absolute freedom from debt and unrestricted receipt of its subscriptions, as provided for in the code of the U. S. Postal Regulations." (Passed without a dissenting voice!)

The above resolution refers entirely to the consultation question! It is to be hoped that delinquent subscribers will *consult* their earliest convenience and pay their dues.

---

### CONTENTS.

**MISCELLANEOUS.** PAGE

The Past and the Future. Editorial, . . . . . . . . . 473

The Law of Cure, the Guide. Carroll Dunham (extract), . . . 473

The London Lancet Describes the Homœopathic Physician (extract), . 474

Plagiarism. Ad. Lippe, M. D., . . . . . . . . . 474

Better After Sleep. C. Hering (extract), . . . . . . . 477

Bœnninghausen's Experience with High Potencies, . . . . . 477

Sulphur and Malaria (extract), . . . . . . . . . 479

Authors of accepted papers are furnished with copies of the number in which their articles appear; application for such copies to be made when sending papers. Any irregularity in the receipt of this Journal should be promptly reported to Editor.

